# Exploring the anti-diabetic potential of banana peel extracts: Impact of maceration and ultrasonication on bioactive compounds and glycemic control in diabetic rabbits

**DOI:** 10.1016/j.ultsonch.2025.107426

**Published:** 2025-06-11

**Authors:** Faiqa Chaudhry, Muhammad Laiq Ahmad, Zafar Hayat, Muhammad Wasim Sajid, Muhammad Mustafa Qamar, Zunaira Basharat, Muhammad Rizwan Tariq, Amna Bibi, Tawfiq Alsulami, Suleiman A Athawab, Robert Mugabi, Basim M. Alohali, Gulzar Ahmad Nayik

**Affiliations:** a Department of Allied Health Sciences, Sargodha Medical College, University of Sargodha, Sargodha 40100, Pakistan; b Department of Animal Sciences, College of Agriculture, University of Sargodha, Sargodha 40100, Pakistan; c Department of Biosciences, COMSATS University Islamabad Sahiwal Campus, Pakistan; d Allied Health Sciences, University of Sargodha, Sargodha 40100, Pakistan; e School of Food & Biological Engineering, Jiangsu University, Zhenjiang, China; f Department of Food Sciences, University of the Punjab, Lahore, Pakistan; g Department of Public Health, University of the Punjab, Lahore, Pakistan; h Department of Food Science & Nutrition, College of Food and Agricultural Sciences, King Saud University, Riyadh 11451, Saudi Arabia; i Department of Food Technology and Nutrition, Makerere University, Kampala, Uganda; j Marwadi University Research Centre, Department of Microbiology, Marwadi University, Rajkot, Gujarat 360003, India

**Keywords:** Banana peel extracts, Sonication, Maceration, Antioxidant, Anti-diabetic, Biochemical markers

## Abstract

Banana peel is an excellent source of fibre and antioxidants. In the current study, extraction parameters for banana peels were optimized using various extraction methods (sonication and maceration), solvents (acetone, ethanol, and methanol) and solvent concentrations (25, 50, 75, 100 %). Furthermore, the antioxidant, antimicrobial and *in-vivo* diabetic potential of banana peel extracts (BPE) was investigated. Based on optimization results, the highest TPC (31.45 mg GAE/g), TFC (22.15 mg QE/g), DPPH (82.52 %), and FRAP (29.51 %) activity was shown by 50 % sonicated ethanolic banana peel extracts (SEBPE). Similarly, 50 % SEBPE showed the highest microbial inhibition zone. However, it was in a dose-dependent manner. The optimum dose was 750 µl/ml for bacterial strains and 500 µl/ml for *S. cerevisiae.* Furthermore, the administration of SEBPE significantly (*p < 0.05*) improved the glycemic indicators in diabetic rabbits compared to control subjects. The highest efficacy was seen in the G5 group, receiving (750 ml of SEBPE/kg body weight) with 205–109 mg/dL and 6.85–4.51 % serum glucose and HbA1c%, respectively, for 30 days. Biochemical markers such as serum protein, albumin, creatinine and lipid profile showed non-significant (*p > 0.05*) variations. However, a significant reduction in total cholesterol (159–90 mg/dL) was attributed to higher fibre. Hence, it can be concluded that sonication improves the antioxidant potential of SEBPE due to the increased release of polyphenols, which are responsible for their enhanced antimicrobial and anti-diabetic properties. However, further investigations are required to explore the effectiveness of SEBPE against diabetes co-morbidities such as renal dysfunction.

## 1 Introduction

Bananas are widely cultivated around the globe, particularly in tropical and subtropical Asian areas; banana production accounted for 116.8 million tonnes in 2020 [1]. In Pakistan, Sindh and Punjab provinces contribute approximately 1.96 million tonnes of banana production annually [2]. Banana is extensively used in culinary applications, being an excellent energy source and high in fiber and micronutrients, leaving enormous amounts of banana peels (BP) as waste. Similarly, BP is a natural reservoir of dietary fibre, micronutrients and polyphenols, contributing to its biological activities such as antioxidant, anti-microbial and anti-diabetic properties [3]. Adopting sustainable approaches, BP as a cost-effective alternate has been studied for various biotechnological application. BP has been incorporated in different food matrices to improve their anti-oxidant activity [4] and nutritional enhancement through fiber supplementation [5]. BP has been utilized for fabrication of different bioplastics films [6] and edible food wrappers [7], enhancing storage stability of products. Furthermore, BP found efficient implications as biofertilizer [8] and biofuel source including bio-ethanol and bio-oil [9,10], aligning with concept of circular economy.

Diabetes mellitus (DM) is the most common metabolic disorder, affecting 2.8 % of the global population, with a projection of a 5.4 % surge by 2025. Additionally, DM exists with co-morbidities, including neuropathy, nephropathy, CVDs and obesity. BPs significantly inhibit the occurrence of oxidative stress-induced metabolic disorders, including T2DM. The anti-diabetic properties of BPs are associated with their dietary fibre and polyphenols, regulating glucose metabolism through modulating glucose absorption, gut microbiota dysbiosis and reversing oxidative damage (Zaini et al. [11]. Zahid et al [12] confirm the administration of BPEs attenuates diabetes-induced renal dysfunction in rabbits. Whereas BP dietary fibre improves the expression of PI3K, AKT, IRS-1, and FOXO1 in hepatic cells of diabetic mice, regulating insulin resistance and insulin signal transduction to mitigate DM [13].

Ultrasound treatment is a sustainable green technology employed to extract phyto-constitutes from plant matrices with higher efficacy and efficiency than conventional extraction approaches. Ultrasound waves induce a cavitation process, disintegrating cellular structure and facilitating the release of polyphenols [14]. Sonication has been extensively used to extract bioactive components from fruit by-products as cavitation increases their release [15]. Furthermore, optimized solvent selection further augments process efficacy. Ethanol is most commonly employed solvent for polyphenols extraction due to higher polyphenol solubility and its GRAS status making it suitable for in-vivo application [16]. Similarly, Tufail et al. [17] reported utilization of methanol and acetone as effective solvents for polyphenols extraction. Previously, BP-polyphenols have been extracted employing maceration, solvent extraction[18], thermal treatments [19] and enzyme-assisted extraction [20]. The Current study focuses on the optimization of extraction conditions of BPE and explores their antioxidant, antibacterial and *in-vivo* anti-diabetic properties.

## 2 Material and methods

### 2.1 Procurement of raw materials

Whole ripe banana *Musa sapientum* (Basrai) was purchased from the local market in Sargodha, Pakistan. The University of Sargodha's laboratories provided all the solvents and reagents used, which were of analytical grade.

### 2.2 Preparation of banana peel powder

The bananas were washed and peeled. The peels were dried with a paper towel to remove excessive water. They were then dried at 40 ℃ for 48 hrs, ground into a fine powder (80 mesh) using a Cyclotec Sample Mill, and stored in an airtight polythene zip bag for further analysis [21].

### 2.3 Determination of the proximate composition of banana peel powder

The proximate composition (moisture, ash, crude protein, crude fat, crude fibre, and NFE) of banana peel powder (BPP) and fresh peels were determined using standard methods of AOAC International as described by Akram et al. [22].

### 2.4 Preparation of banana peel extracts (BPEs)

#### 2.4.1 Maceration-assisted extraction (MAE)

BPP was macerated with acetone, methanol, and ethanol at solvent concentrations of 25 %, 50 %, 75 %, and 100 % with a sample: solvent weight ratio of 1:15. It was then vortexed and placed in a water bath at 25 °C for 24 hrs with continues agitation at 150 rpm. Subsequently, filtered and centrifuged at 5000 rpm for 10 min. The supernatant (BPE) was extracted from centrifuge tubes, transferred to separate storage vials, and stored at 4 °C for further analysis.

#### 2.4.2 Sonication-assisted extraction (SAE)

BPP extracted with acetone, methanol, and ethanol at solvent concentrations of 25 %, 50 %, 75 %, and 100 % with a sample: solvent ratio of 1:15. It was then vortexed and placed in an ultrasonic bath (Model 6.51200H) operated at 40KHz frequency at 30 °C for 30 min. Afterwards, it was centrifuged at 5000 rpm for 10 min. The supernatant (BPE) was extracted from centrifuge tubes, transferred to separate storage vials, and stored at 4 °C for further analysis.

### 2.5 Determination of antioxidant potential of BPEs

#### 2.5.1 Determination of total phenolic content (TPC)

The TPC of BPE was determined using the Folin-Ciocalteu method as described by Semangoen *et al.* [11] with few modifications. Briefly, 10 μL of the sample (BPE) was mixed with 25 μL of FC-reagent and 40 μL of 7.5 % sodium carbonate, vortexed, and left in the dark for 30 mins. Absorbance was measured at 765 nm in a Spectrophotometer, and TPC was expressed as mg gallic acid equivalents (GAE) per 100  mL of sample.

#### 2.5.2 Determination of total flavonoid content (TFC)

TFC was determined using the method of Kamal *et al.* [23] with few modifications. Briefly, the solution consisted of 0.5 ml extract combined with 2 ml distilled water before the addition of 0.15 ml NaNO_2_ 5 %, followed immediately by 0.15 ml 10 % AlCl_3_ after 5 min, and allowed to rest in the dark for 15 min. Afterwards, absorbance was measured at 510 nm using a spectrophotometer (UV-1800, Shimadzu Corporation, Kyoto, Japan). TFC was expressed as mg quercetin equivalent per 100 mL of the sample.

#### 2.5.3 Determination of DPPH radical scavenging activity

The DPPH free radical scavenging activity was determined following the method of Hasan *et al.* [24]. Briefly, 25 µl extract was mixed with 1.5 ml DPPH methanolic solution and allowed to rest in the dark for 15 mins at room temperature. Later on, absorbance was taken at 515 nm using a spectrometer.

$$\%Inhibition=Ac-\frac{\left(As-Ac\right)}{\mathrm{Ac}}\times 100$$

Whereas:

Ac: absorbance capacity of solution without DPPH.

As: absorbance capacity of solution with DPPH.

*Determination of FRAP activity*.

FRAP activity of samples was assessed using the method of Yaqoob et al. [25] with few modifications. Briefly, 0.6 ml of FRAP solution was homogenized with 2 ml of sample and 0.6 ml water and allowed to react at 37C in a water bath for 30 min. Afterwards, absorbance was taken at 595 nm, and results were calculated as mg of Trolox equivalent/mL of sample.

### 2.6 Determination of the anti-microbial potential of BPEs

The antimicrobial potential of BPEs against *S. aureus*, *P. aeruginosa*, *E. coli*, and *S. cerevisiae* was evaluated using the disc diffusion method described by Kapoor *et al.* [26]. Briefly, tubes containing agar with seeded microbial cultures were placed on top of the agar surface, and a reservoir of BPE was placed on top of the agar surface. BPE was diffused into the agar, and inhibition zones were measured after incubation.

### 2.7 Evaluation of the in-vivo antidiabetic potential of sonicated ethanolic banana peel extract (SEBPE)

#### 2.7.1 Rabbits' diet and management

Rabbits (4 months old, 1.5–2.5 kg) of albino genotype were purchased and housed under controlled temperature (33 ± 1°C) and light (12/24 h). The rabbits were fed a standard diet with 20 % protein, 10 % sucrose, 4 % soybean oil, 2 % choline chloride, 3.5 % salt mixture, 1 % vitamin mixture, 5 % fibre, and the remaining composition was 100 % corn starch. The diet was planned and prepared at the food microbiology laboratory at the University of Sargodha to maintain the blood glucose level. Before the experiment, the rabbits were adjusted for two weeks.

#### 2.7.2 Diabetes induction in subjects

Diabetes was induced by injecting alloxan dissolved in saline (90 ml/kg body weight). After 72 h, subjects were investigated for diabetes in a fasting state. As hyperglycemia was confirmed, subjects were weighed and subjected to treatment.

#### 2.7.3 Experimental design and treatment groups

The experimental design is presented in Table 1, detailing the treatment groups (n = 6), administered dosages, and intervention plans. Rabbits were divided into five groups, each receiving specific treatments to evaluate the effects of SEBPE on glycemic control and metabolic health. The control group was fed a standard diet without any intervention, while the negative control group was diabetic but received no treatment. Other groups received different concentrations of SEBPE, allowing a comparative assessment of its impact on glucose regulation and biochemical markers. Treatments were administered daily, and changes in metabolic parameters were monitored over 30 days.

**Table 1:** Experimental design for determination of *in-vivo* anti-diabetic potential of sonicated ethanolic banana peel extracts.

**Groups** **(n = 06)**	**Coding**	**Treatment**	**Administration dosage**
**Group-I**	**G-1**	Control	Feed and distilled water
**Group-II**	**G-2**	Negative control	Diabetics induced rabbits with no treatment
**Group-III**	**G-3**	SEBPE	250 ml/kg ethanolic extract
**Group-IV**	**G-4**		500 ml/kg ethanolic extract
**Group-V**	**G-5**		750 ml/kg ethanolic extract

SEBPE: Sonicated 50% ethanolic banana peel extract.

#### 2.7.4 Blood sample collection

Before sample collection, the rabbits fasted overnight with free access to water. Blood glucose levels were measured using a glucometer and collected from the *retro*-orbital plexus. Blood was drawn into 5 mL tubes containing EDTA for serum separation. The serum was centrifuged at 14,000 rpm for 5 min and used for subsequent analysis.

#### 2.7.5 Determination of weight gain

Subjects' Body weight was measured using a digital weighing balance at the start of the study and recorded in grams. Afterwards, readings were taken on the 8th, 15th, and 29th day of the study to observe any risks of obesity.

#### 2.7.6 Determination of serum glucose

Subjects' serum glucose levels were measured using a digital glucometer provided by The Contour Next EZ Blood Glucose Monitoring System (New Jersey, USA), following the method of Liang et al. [27].

#### 2.7.7 Determination of HbA1c

The A1C Now + test system (PTS Diagnostics, USA) assessed haemoglobin A1c (HbA1c). The blood sample was placed in a cartridge containing an anti-HbA1c antibody and allowed to react under gentle shaking. Afterwards, the cartridge was loaded, and the results were recorded as %A1c [27].

### 2.8 Analysis of serum biochemical markers of diabetic rabbits

#### 2.8.1 Determination of serum total proteins and albumin

Serum total proteins and serum albumin were measured using chemical kits (Biolabo, France), following the method of Abduljabbar et al.[28].

#### 2.8.2 Determination of serum triglycerides (TG) and serum cholesterol (TC)

Serum triglycerides and cholesterol were evaluated using the GPO-PAP and CHOD-PAP methods, respectively, as described by Rizwan Tariq et al. [29].

#### 2.8.3 Determination of high-density lipoprotein (HDL) and low-density lipoprotein (LDL)

Blood serum HDL was measured using an HDL-cholesterol assay kit as described by Rizwan Tariq et al. [29]. Briefly, 100 µL of serum was mixed with 250 µL of diluted reagent and kept at room temperature for 10 min. Afterwards, absorbance was measured at 546 nm using a spectrophotometer (CESIL CE7200, England). Whereas blood serum LDL was determined following the method of Abduljabbar et al. [28] and measured using the following equation;

$$LDL\left(\frac{\mathrm{mg}}{\mathrm{dL}}\right)=Totalcholestrol-\left(HDL+\frac{\mathrm{Triglycerides}}{5}\right)$$

#### 2.8.4 Determination of creatinine levels

Serum creatinine levels were determined using Jaffe's calorimetric method, as described by Delanghe and Speeckaert [30]. The blood sample was allowed to react with picric acid in a basic condition, and then absorbance was recorded through a spectrometer.

### 2.9 Statistical analysis

The obtained data for each parameter were subjected to statistical analysis using Minitab 18.1. Results were presented as means and standard deviations, and ANOVA was applied to determine the significance level, followed by LSD, confidence interval was 95 %. All the experiments were performed in triplicate and.

## 3 Results & discussion

### 3.1 Proximate composition of fresh and dried banana peels

The proximate composition of fresh and dried banana peels is presented in Table 2. It is evident from Table 2 that moisture reduction increases the nutrient densities in dried peel powder compared to fresh peel powder. A significant increase was noticed in the protein (2.52 ± 0.24–5.82 ± 0.11) and fibre content (6.36 ± 0.04–23.19 ± 0.27) of fresh and dried peel powders. Dehydration improves the stability of peel powder and enhances its nutritional profile, making it a potential source of fibre supplementation in functional foods with considerable amounts of protein and minerals. Similarly, Cunha *et al.* [31] and Wetmore *et al.* [32] reported significant variations in the biochemical composition of fresh and dried mango and carrot skins, respectively.

**Table 2:** Proximate composition of fresh and dried banana peels.

**Variables (g/100 g)**	**Fresh**	**Dried**
Moisture	55.48 ± 0.67	10.87 ± 0.04
Protein	2.52 ± 0.24	5.82 ± 0.11
Fat	0.60 ± 0.47	2.87 ± 0.26
Carbohydrates	28.42 ± 0.21	44.77 ± 0.19
Crude Fiber	6.36 ± 0.04	23.19 ± 0.27
Ash content	6.62 ± 0.11	12.48 ± 0.39

### 3.2 Antioxidant potential of banana peel extracts (BPEs)

#### 3.2.1 Total phenolics content and total flavonoids in BPEs

Polyphenols such as phenolics and flavonoids are essential phytochemicals, possessing vigorous antioxidant activity contributing to BPE's therapeutic and functional characteristics. TPC and TFC of BPE prepared using different concentrations of acetone, ethanol and methanol were determined. Our results showed that all three organic solvents were suitable for extracting phenolics and flavonoids. However, the highest TFC and TPC were found in 75 % acetone, 50 % ethanol and 50 % methanol extracts for macerated and sonicated extracts, as shown in Table 2. In comparison to macerated extracts, there was a significant (*p < 0.05*) increase in TPC of sonicated acetone (20.85–23.81 mg GAE/g), ethanol (27.35–31.45 mg GAE/g) and methanol (23.50–28.13 mg GAE/g) extracts of banana peels. Similarly, TFC was higher in sonicated acetone, ethanol and methanol extract of banana peels at 19.83, 22.15 and 20.86 mg QE/g, respectively. It is evident from Table 3 that the highest yield of TPC (31.45 mg GAE/g) and TFC (22.15 mg QE/g) was obtained by 50 % sonicated ethanolic extracts. Sonication enhances the release of bioactive components by disrupting plant cell walls and transforming bound polyphenols into free compounds. Our results are based on a study by Granella *et al.* ([33]) that found that ultrasound treatment improves the recovery rate from banana peel extracts. Similarly, Tufail *et al.*[34] reported higher yields of TPC and TFC in US-treated barley flours.

**Table 3:** Optimization of banana peel extracts prepared using different solvent at various solvents concentration by evaluating their anti-oxidant potential.

**Extraction Method**	**Solvent**	**Solvent Concentration**	**TPC** (mg GAE/g)	**TFC** (mg QE/g)	**DPPH** (%)	**FRAP** (%)
**Sonicated assisted extraction**	**Acetone**	25 %	18.66 ± 1.5^d^	15.15 ± 0.5^c^	65.69 ± 2.1^d^	21.26 ± 0.7^d^
		50 %	20.21 ± 1.7^b^	16.06 ± 0.3^b^	73.80 ± 2.5^b^	26.02 ± 0.7^b^
		75 %	23.81 ± 1.5^a^	19.83 ± 0.5^a^	76.71 ± 2.3^a^	27.52 ± 0.3^a^
		100 %	18.29 ± 1.8^c^	13.87 ± 0.6^d^	66.68 ± 2.3^c^	22.91 ± 0.4^c^
	**Ethanol**	25 %	21.03 ± 1.7^d^	16.28 ± 0.5^d^	76.40 ± 0.5^c^	25.11 ± 0.2^d^
		50 %	31.45 ± 1.7^a^	22.15 ± 0.5^a^	82.52 ± 0.3^a^	29.51 ± 0.6^a^
		75 %	27.19 ± 1.8^b^	20.11 ± 0.5^b^	79.98 ± 0.5^b^	27.56 ± 0.8^c^
		100 %	24.21 ± 1.5^c^	17.47 ± 0.4^c^	74.17 ± 0.4^d^	26.95 ± 0.2^b^
	**Methanol**	25 %	19.63 ± 1.4^d^	17.75 ± 0.6^c^	65.97 ± 0.3^b^	22.82 ± 2.4^d^
		50 %	28.13 ± 1.5^a^	20.86 ± 0.5^a^	72.65 ± 0.3^a^	28.36 ± 2.3^a^
		75 %	23.18 ± 1.5^b^	19.44 ± 0.6^b^	60.88 ± 0.6^c^	24.95 ± 2.5^b^
		100 %	20.02 ± 1.3^c^	15.43 ± 0.6^d^	70.33 ± 0.5^d^	25.21 ± 2.3^c^
**Maceration assisted extraction**	**Acetone**	25 %	15.45 ± 1.8^c^	12.24 ± 0.2^c^	63.46 ± 2.5^d^	18.59 ± 2.3^d^
		50 %	17.95 ± 1.6^b^	13.01 ± 0.3^b^	66.82 ± 2.7^b^	20.96 ± 0.2^c^
		75 %	20.85 ± 1.5^a^	15.77 ± 0.5^a^	72.75 ± 2.5^a^	23.65 ± 0.4^a^
		100 %	15.98 ± 1.4^c^	10.75 ± 0.5^d^	64.01 ± 2.3^c^	21.81 ± 0.3^b^
	**Ethanol**	25 %	20.85 ± 1.5^c^	15.42 ± 0.6^c^	70.12 ± 0.6^c^	21.18 ± 0.5^c^
		50 %	27.35 ± 1.5^a^	18.01 ± 0.5^b^	78.85 ± 0.5^a^	25.31 ± 0.4^a^
		75 %	19.62 ± 1.8^d^	19.07 ± 0.4^a^	72.78 ± 0.5^b^	23.33 ± 0.5^b^
		100 %	21.10 ± 1.2^b^	15.60 ± 0.3^c^	69.92 ± 0.7^d^	21.58 ± 0.3^c^
	**Methanol**	25 %	17.53 ± 1.5^c^	14.22 ± 0.9^c^	64.86 ± 0.2^d^	18.38 ± 0.4^d^
		50 %	23.50 ± 1.3^a^	17.33 ± 0.9^a^	68.48 ± 0.2^a^	24.17 ± 0.4^a^
		75 %	21.79 ± 1.3^b^	16.37 ± 0.4^b^	66.37 ± 0.5^c^	20.57 ± 0.2^b^
		100 %	17.98 ± 1.7^c^	13.32 ± 0.6^d^	67.13 ± 0.5^b^	19.45 ± 0.3^c^

Mean values ± SD with different letters (a–d) in individual solvent (4 rows each) are significantly different (p < 0.05).

#### 3.2.2 DPPH & FRAP radical scavenging activity of BPEs

Polyphenols like phenolics and flavonoids are potent antioxidants that help minimize oxidative stress. DPPH and FRAP assays were performed to evaluate the antioxidant capacity at specific acetone, ethanol, and methanol concentrations. The highest antioxidant potential was observed in

75 % acetone, 50 % ethanol, and 50 % methanol extracts for both extraction methods. It is evident from Table 3 that DPPH radical scavenging activity was higher in sonicated extracts as compared to macerated extracts. There was an increase up to 76.71 %, 82.52 %, and 72.65 % for acetone, ethanol and methanol-based sonicated extracts, respectively. Similarly, FRAP activity was also higher in sonicated acetone (21.26–27.52 %), ethanol (25.11–29.51 %), and methanol (22.82–28.36 %) extracts. Among all extracts, 50 % of sonicated ethanolic extracts retained the highest antioxidant capacity, possibly attributed to the higher solubility of banana peel polyphenols in ethanol than other solvents [35]. Hence, the antioxidant potential of banana peels is interlinked with the incidence of polyphenols, contributing to their health-endorsing properties [32]. Previously, Tufail et al. [36] and Yaqoob et al. [37] have reported that ultrasound treatment significantly increased polyphenolic compounds in cereals and mulberry juice. Similarly, Mehmood et al. [38] reported increased anti-oxidant activities in ultrasonicated mulberry leaves. The enhancement in DPPH and FRAP activities are interlinked to enhanced TPC and TFC resulted from ultrasound induced cavitation facilitating membrane breakdown and release of phytochemicals (Basharat et al. [39].

### 3.3 Antimicrobial potential of BPEs

**Fig. 1:**
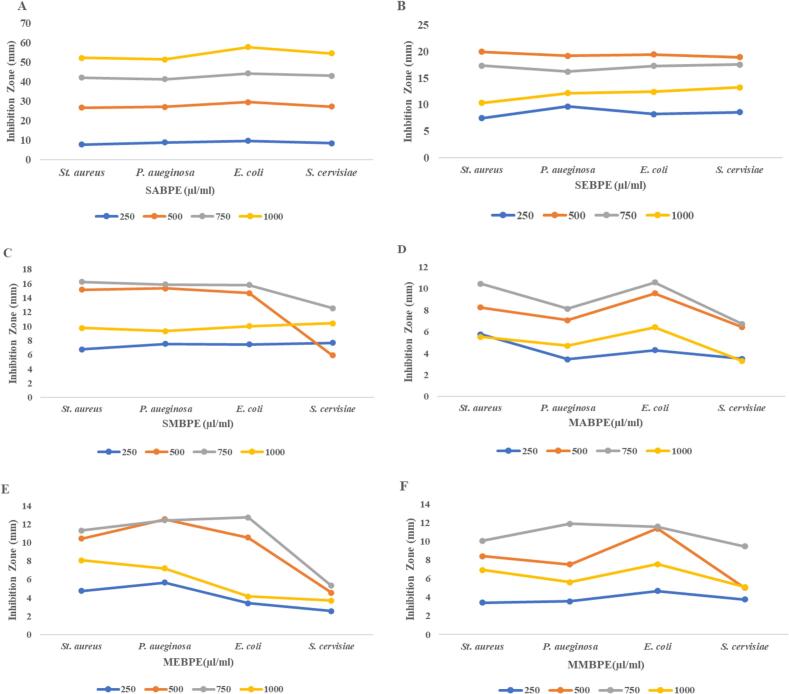
Dose response curves (A) Sonication assisted 75% acetone banana peel extract (B) Sonication assisted 50% ethanolic banana peel extract (C) Sonication assisted 50% methanolic banana peel extract (D) Macerated 75% acetone banana peel extract (E) Macerated 50% ethanolic banana peel extract (F) Macerated 50% methanolic banana peel extract.

The anti-microbial potential of BPE at various concentrations against *S. aureus P. aeruginosa E. coli* and *S. cerevisiae* is elucidated in Table 4. Our results showed that sonication significantly enhanced the anti-microbial potential of BPE compared to macerated extracts. Furthermore, ethanolic extracts retained better inhibiting capacity against microbial strains attributed to their strong antioxidant potential and better retention of polyphenols. However, the anti-microbial activity of BPE function in dose-dependent manner presented in Fig. 1 (A-F). Furthermore, it evident from Table 4, the inhibiting zone was maximum for all bacterial strains at 750 µl/ml, whereas for *S. cerevisiae*, it was maximum at 500 µl/ml. Further increase (1000 µl/ml) in inoculation concentration reduced the inhibition zone for all microbial strains. In the case of plant bioactive, extraction technique and selected solvent have considerable influence on the anti-microbial potential of extracts. Previously, W. Safdar *et al.* [40] and Aboul-Enein *et al.* [41] observed a strong antimicrobial effect of banana peel and orange rind extracts. Similar to our results, Andrade et al. [42] reported efficacy of UAE grape skin extract against *E.coli* and *S.aureus*. Furthermore, pomegranate powder and goji berries ethanol and aqueous extracts were prepared using ultrasound assisted extraction and accessed for their efficacy against various gram positive, gram negative and fungal stains. The results indicated maximum antibacterial efficacy in UAE goji berry extracts whereas UAE pomegranate extracts exhibited both anti-bacterial and anti-fungal properties, attributed to increased polyphenols in US treated extracts [43].

**Table 4:** Optimization of banana peel extracts prepared using different solvent at various solvents concentration by evaluating their anti-microbial activity.

**Banana peel extract**	**Conc. µl/ml**	**Inhibition Zone (mm)**			
		***St. aureus***	***P. aueginosa***	***E. coli***	***S. cervisiae***
**SABPE**	250	7.74 ± 0.04^d^	8.75 ± 0.04^d^	9.59 ± 0.05^d^	8.39 ± 0.03^d^
	500	18.92 ± 0.04^a^	18.24 ± 0.02^a^	19.87 ± 0.04^a^	18.76 ± 0.04^a^
	750	15.35 ± 0.03^b^	14.32 ± 0.02^b^	14.76 ± 0.0^b^	15.89 ± 0.0^b^
	1000	10.27 ± 0.03^c^	10.07 ± 0.03^c^	13.52 ± 0.05^c^	11.41 ± 0.02^c^
**SEBPE**	250	7.45 ± 0.04^d^	9.67 ± 0.05^d^	8.22 ± 0.03^d^	8.56 ± 0.02^d^
	500	19.95 ± 0.04^a^	19.23 ± 0.05^a^	19.45 ± 0.03^a^	18.96 ± 0.02^a^
	750	17.35 ± 0.02^b^	16.25 ± 0.02^b^	17.29 ± 0.05^b^	17.56 ± 0.04^b^
	1000	10.31 ± 0.05^c^	12.19 ± 0.04^c^	12.43 ± 0.04^c^	13.24 ± 0.03^c^
**SMBPE**	250	6.78 ± 0.05^d^	7.54 ± 0.04^d^	7.45 ± 0.03^d^	7.67 ± 0.02^c^
	500	15.15 ± 0.04^b^	15.32 ± 0.03^b^	14.66 ± 0.04^b^	5.92 ± 0.02^d^
	750	16.25 ± 0.04^a^	15.89 ± 0.02^a^	15.81 ± 0.02^a^	12.56 ± 0.04^a^
	1000	9.76 ± 0.03^c^	9.32 ± 0.02^c^	10.01 ± 0.02^c^	10.43 ± 0.04^b^
**MABPE**	250	5.78 ± 0.01^c^	3.45 ± 0.06^d^	4.29 ± 0.02^d^	3.49 ± 0.06^b^
	500	8.27 ± 0.01^b^	7.07 ± 0.04^b^	9.57 ± 0.02^b^	6.45 ± 0.05^a^
	750	10.47 ± 0.03^a^	8.13 ± 0.04^a^	10.57 ± 0.03^a^	6.75 ± 0.05^a^
	1000	5.53 ± 0.03^c^	4.72 ± 0.05^c^	6.42 ± 0.04^d^	3.31 ± 0.04^b^
**MEBPE**	250	4.76 ± 0.04^d^	5.67 ± 0.04^c^	3.42 ± 0.05^d^	2.56 ± 0.01^d^
	500	10.45 ± 0.04^b^	12.56 ± 0.04^a^	10.57 ± 0.05^b^	4.55 ± 0.01^b^
	750	11.32 ± 0.03^a^	12.43 ± 0.03^a^	12.76 ± 0.04^a^	5.32 ± 0.03^a^
	1000	8.07 ± 0.02^c^	7.21 ± 0.05^b^	4.15 ± 0.04^c^	3.71 ± 0.02^c^
**MMBPE**	250	3.42 ± 0.08^d^	3.56 ± 0.04^d^	4.67 ± 0.03^c^	3.76 ± 0.02^c^
	500	8.42 ± 0.08^b^	7.53 ± 0.02^b^	11.36 ± 0.02^a^	5.02 ± 0.04^b^
	750	10.05 ± 0.06^a^	11.89 ± 0.02^a^	11.56 ± 0.02^a^	9.45 ± 0.04^a^
	1000	6.92 ± 0.06^c^	5.62 ± 0.04^c^	7.56 ± 0.06^b^	5.12 ± 0.03^b^

SABPE: Sonication assisted 75% acetone banana peel extract; SEBPE: Sonication assisted 50% ethanolic banana peel extract; SMBPE: Sonication assisted 50% methanolic banana peel extract; MABPE: Macerated 75% acetone banana peel extract; MEBPE: Macerated 50% ethanolic banana peel extract; MMBPE: Macerated 50% methanolic banana peel extract.

Mean values ± SD with different letters (a–d) in individual parameter (4 rows each) are significantly different (p < 0.05).

### 3.4 In-vivo anti-diabetic potential of sonicated ethanolic banana peel extract (SEBPE)

#### 3.4.1 Effect of SEBPE on glycemic response indicators of diabetic rabbits

Diabetes is the most frequent lifestyle related disorder that is associated with various metabolic disorders. The effect of BPE administration was evaluated on diabetic markers such as weight gain, serum glucose and HbA1c in alloxan-induced rabbits. The results depicted in Table 5 showed improved glycemic response in SEBPE-administrated rabbits compared to control subjects. Diabetes is the root cause of obesity and weight gain; as compared to both controls, there was a significant decrease in weight gain in SEBPE-treated rabbits. There was a drastic increase in weight for both G1 (1.35 g-2.48 g) and G2 (2.35–4.81 g). However, in SEBPE-treated groups, the weight gain was gradual and low; among them, the lowest increase (0.65–0.97 g) was observed in G5, treated with 750 ml/kg SEBPE. BPE improves energy utilization by activating heat-production to maintain an optimal metabolic state [44]. According to Kokate et al. [45], banana peel supplementation is a practical weight management approach in obese people.

**Table 5:** Effect of sonicated ethanolic extracts of banana peels on diabetic markers of diabetic rabbits for 30 days.

**Diabetic markers**	**Days**	**Control**	**Negative control**	**SEBPE**		
		**G-1**	**G-2**	**G-3**	**G-4**	**G-5**
**Weight** **Gain** (g)	**0**	**−**	**−**	**−**	**−**	**−**
	**8**	1.35 ± 0.5^d^	2.35 ± 0.02^b^	0.67 ± 0.02^f^	0.66 ± 0.1^f^	0.65 ± 0.1^f^
	**15**	2.14 ± 0.5^bc^	3.99 ± 0.2^a^	1.28 ± 0.03^d^	0.98 ± 0.2^e^	0.82 ± 0.2^ef^
	**29**	2.48 ± 0.5^b^	4.81 ± 0.03^a^	1.89 ± 0.03^bc^	1.11 ± 0.3^de^	0.97 ± 0.2^e^
**Serum** **Glucose** (mg/dL)	**0**	99.40 ± 0.04^h^	205.80 ± 0.3^a^	206.20 ± 0.2^a^	204.80 ± 0.02^a^	205.10 ± 0.2^a^
	**8**	102.60 ± 0.04^h^	198.50 ± 0.5^a^	187.40 ± 0.1^b^	180.50 ± 0.2^bc^	152.80 ± 0.04^d^
	**15**	92.80 ± 0.2^h^	199.30 ± 0.5^a^	162.80 ± 0.2^cd^	141.90 ± 0.2^e^	126.90 ± 0.3^f^
	**29**	94.50 ± 0.2^h^	21090 ± 0.2^a^	155.10 ± 0.0^d^	137.20 ± 0.02^e^	109.50 ± 0.3^gh^
**HbA1c** (%)	**0**	4.65 ± 0.3^e^	6.87 ± 0.2^bc^	6.94 ± 0.2^b^	6.78 ± 0.5^bc^	6.85 ± 0.1^bc^
	**8**	4.62 ± 0.2^e^	7.84 ± 0.4^ab^	6.16 ± 0.3^cd^	6.18 ± 0.03^cd^	5.96 ± 0.2^cd^
	**15**	4.58 ± 0.1^e^	8.35 ± 0.3^a^	5.93 ± 0.1^cd^	5.95 ± 0.5^cd^	5.38 ± 0.3^d^
	**29**	4.59 ± 0.2^e^	9.18 ± 0.1^a^	5.33 ± 0.4^d^	5.41 ± 0.5^d^	4.51 ± 0.4^e^

SEBPE: 50% sonicated ethanolic banana peel extract.

Mean values ± SD with different letters (a–h) in individual parameter (4 rows each) are significantly different (p < 0.05).

Table 5 shows that SEBPE markedly improved serum glucose levels in diabetic rabbits compared to the control. However, the lowest serum glucose levels were noticed in G5 (205.10–109.50 mg/dL). Furthermore, in the control group (205.80–210.90 mg/dL), diabetes progressed over time, whereas in the treated rabbits, there was a significant reduction in glucose levels (205.10–109.50 mg/dL).

A similar trend was observed for HbA1c in control and treated rabbits, as SEBPE significantly improves HbA1c % in diabetic rabbits. Initially, HbA1c was 6.87 % decreased to 4.51 % in the G5 group. The results showed that administration of SEBPE improves diabetic markers and lessens the adversity of diabetes. However, administration dose plays a crucial role in metabolism regulation. The findings regarding HbA1c and serum glucose reduction align with [46], which reported the anti-diabetic properties of fruit peels. Similarly, Bagabaldo *et al.* [47] stated that supplementation of fruit peels reduces HbA1c levels to achieve glycaemic stability.

#### 3.4.2 Effect of SEBPE on serum biochemical markers of diabetic rabbits

Serum biochemical markers are essential indicators of normal body functioning and metabolic health. The effect of SEBPE on serum proteins, lipid profile and creatinine levels were evaluated to determine the efficacy of BPE against diabetes and its related disorders. The results showed that compared to G1 (3.48 mg/dL), serum protein was increased in diabetic rabbits (4.92–5.51 mg/dL). However, Table 6 indicated a significant decrease in treated subjects compared to G2. However, for all subjects, the serum protein didn't fall in the normal range, possibly related to improper absorption of proteins. Furthermore, lower protein levels could be linked to diabetes-induced renal issues.

**Table 6:** Effect of sonicated ethanolic extracts of banana peels on biochemical markers of diabetic rabbits for 30 days.

**Biochemical markers** **(mg/dL)**	**Days**	**Control**	**Negative control**	**SEBPE**		
		**G-1**	**G-2**	**G-3**	**G-4**	**G-5**
**Total serum protein**	**0**	3.48 ± 0.5^de^	5.51 ± 0.7^c^	5.39 ± 0.5^c^	5.04 ± 0.5^c^	4.92 ± 0.5^cd^
	**8**	3.19 ± 0.5^e^	6.21 ± 0.5^c^	4.34 ± 0.2^d^	4.16 ± 0.5^d^	3.75 ± 0.4^e^
	**15**	3.31 ± 0.4^e^	6.96 ± 0.5^b^	4.42 ± 0.2^d^	4.32 ± 0.3^d^	3.63 ± 0.4^ef^
	**29**	3.05 ± 0.3^e^	7.49 ± 0.3^a^	3.49 ± 0.4^d^	3.43 ± 0.4^d^	2.38 ± 0.3^f^
**Serum albumin**	**0**	3.44 ± 0.6^e^	3.21 ± 0.3^f^	3.37 ± 0.5^e^	3.35 ± 0.2^e^	2.87 ± 0.5
	**8**	3.52 ± 0.5^d^	3.35 ± 0.2^e^	3.75 ± 0.5^bc^	3.69 ± 0.2^cd^	3.75 ± 0.5^bc^
	**15**	3.49 ± 0.6^de^	3.32 ± 0.2^e^	3.79 ± 0.4^bc^	3.71 ± 0.5^c^	4.25 ± 0.6^a^
	**29**	3.82 ± 0.6^b^	3.15 ± 0.3^ef^	3.8 ± 0.4^b^	3.75 ± 0.5^bc^	4.41 ± 0.6^a^
**Serum triglycerides**	**0**	86.73 ± 0.3^f^	193.25 ± 0.2^a^	193.77 ± 0.5^a^	197.29 ± 0.2^a^	181.81 ± 0.7^a^
	**8**	88.1 ± 0.3^f^	185.8 ± 0.2^a^	162.5 ± 0.5^b^	157.2 ± 0.2^bc^	136.9 ± 0.7^cd^
	**15**	81.5 ± 0.6^f^	189.6 ± 0.2^a^	146.7 ± 0.4^c^	127.8 ± 0.2^d^	104.9 ± 0.5^e^
	**29**	85.4 ± 0.5^f^	194.17 ± 0.4^a^	127.94 ± 0.4^d^	109.71 ± 0.5^de^	86.48 ± 0.5^f^
**HDL**	**0**	31.3 ± 0.25^fhij^	31.3 ± 0.15^hj^	30 ± 0.10^ij^	30 ± 0.65^ij^	28.3 ± 0.55^jk^
	**8**	33.3 ± 0.15^fghi^	27.6 ± 0.11^jk^	35.6 ± 0.25^g^	36.6 ± 0.54^fg^	46 ± 0.25^fg^
	**15**	34.6 ± 0.23^fgh^	27.3 ± 0.18^jk^	40 ± 0.13^ef^	44.6 ± 0.21^bcd^	46 ± 0.12^ab^
	**29**	33.3 ± 0.14^fgh^	24.6 ± 0.11*^k^*	45 ± 0.56^bcd^	47 ± 0.36^ab^	50.33 ± 0.15^a^
**LDL**	**0**	121.3 ± 0.5^bc^	120.3 ± 0.4^bc^	119 ± 0.8^c^	121.3 ± 0.2^bc^	119 ± 0.7^c^
	**8**	121.3 ± 0.5^bc^	124 ± 0.2^b^	110.6 ± 0.3^d^	105 ± 0.5^ef^	100.6 ± 1.0^fg^
	**15**	122.6 ± 0.3^bc^	133 ± 0.3^a^	95 ± 0.3^hi^	92 ± 0.5^i^	86 ± 0.8^j^
	**29**	121.6 ± 0.5^bc^	132 ± 0.2^a^	80 ± 0.5^k^	75 ± 0.2^l^	71.6 ± 0.7^k^
**Total cholesterol**	**0**	160 ± 0.05^b^	158 ± 0.07^b^	159 ± 0.05^b^	160 ± 0.25^b^	159 ± 0.12^b^
	**8**	161 ± 0.03^b^	161 ± 0.05^b^	140 ± 0.05^d^	135 ± 0.03^e^	130 ± 0.13^f^
	**15**	158 ± 0.03^b^	168 ± 0.05^a^	125 ± 0.02^g^	110 ± 0.03^j^	110 ± 0.12^j^
	**29**	157 ± 0.02^b^	170 ± 0.03^a^	110 ± 0.03^j^	90 ± 0.06^l^	90 ± 0.15*^m^*
**Creatinine**	**0**	0.9 ± 0.05^c^	1.3 ± 0.06^a^	1.3 ± 0.04^a^	1.3 ± 0.03^a^	1.4 ± 0.05^a^
	**8**	0.7 ± 0.05^d^	1.4 ± 0.02^a^	1.2 ± 0.04^b^	1.1 ± 0.05^bc^	0.9 ± 0.05^c^
	**15**	0.7 ± 0.03^d^	1.2 ± 0.02^b^	1.1 ± 0.02^bc^	1.1 ± 0.05^bc^	0.7 ± 0.02^d^
	**29**	0.8 ± 0.02^cd^	1.5 ± 1.0^a^	1.1 ± 0.02^bc^	1.1 ± 0.05^bc^	0.7 ± 0.02^d^

SEBPE: 50% sonicated ethanolic banana peel extract.

Mean values ± SD with different letters (a–j) in individual parameter (4 rows each) are significantly different (p < 0.05).

Serum albumin (SA) showed a heterogenous trend in diabetic control (G2) and treated rabbits compared to G1. Furthermore, it is evident from Table 6 that increased concentration of SEBPE significantly reduced (3.37–2.87 mg/dL) SA in diabetic rabbits. However, with the progression of time, SA increased for all subjects, but it was highest in G5 (2.87–4.41 mg/dL) compared to diabetic control (3.21–3.15 mg/dL). Our findings are by Chaudhry *et al.*, [35] suggested the efficacy of BPE therapy in boosting albumin concentration in diabetic rabbits and indicated improved hepatic function protein metabolism.

The results showed a non-significant variation in the lipid profile of diabetic rabbits on the administration of SEBPE compared to the control. However, over time, the decrease was highest (181–86 mg/dL) in G5. Similarly, a non-significant variation was observed in the HDL and LDL levels of diabetic rabbits. Initially, non-significant variation was observed in total serum cholesterol (TC) of all subjects; however, at the end of the study, there was a significant decline in TC (160–90 mg/dL) of diabetic rabbits as compared to diabetic control (158–170 mg/dL). The reduction in TC was, in a dose-dependent manner, beginning highest in G5 (159–90 mg/dL), receiving 750 mg/kg SEBPE attributed to higher fibre levels in BPE, increasing its cholesterol binding capacity. Similarly, reduced triglyceride levels [45] and regulated lipid profiles have been observed in experimental subjects who received banana peel supplementation [48].

Furthermore, the results showed a higher incidence of creatinine levels in diabetic rabbits (1.3–1.5 mg/dL) as compared to the control (0.7–0.9 mg/dL). However, in treated subjects, creatinine levels decreased (1.3–0.7 mg/dL) non-significantly in comparison to G2 (1.5–1.3 mg/dL). The increased creatinine levels in diabetic rabbits could be an indicator of diabetic-induced impaired renal function requiring further investigations.

## 4 Conclusions

Banana peels are a rich natural source of dietary fibre and bioactive compounds such as antioxidants. Polyphenols are relevant and responsible for their techno-functional and therapeutic properties. In alignment with sustainable goals, adopting circular economy approach for BP is imperative, constituting 30–40 % of total fruit waste. Their valorization reduces landfill burdens but also enhance resource efficiency. Furthermore, pilot scale sonication achieves 80 % yield of bioactive compounds with higher efficiency over conventional approaches while maintaining economic viability. In this context, we have optimized extraction conditions for banana peel extracts using maceration and sonication at various concentrations (25 %, 50 %, 75 %, 100 %) of different organic solvents (acetone, ethanol, and methanol). 50 % SEBPE showed the best antioxidant and anti-microbial potential. SSEBPE were further investigated for anti-diabetic properties in alloxan-induced diabetic rabbits. Results suggested improved glycemic response and reduced total cholesterol; however, for our study, serum proteins, total glycerides, and creatinine levels showed non-significant variations in response to SEBPE administration requiring further studies diabetes-induced co-morbidities. The future studies could be focused on further optimization of extraction conditions or dose escalation, long term effect of SEBPE in animal models or transcriptomic studies on renal genes. However, the optimized extraction conditions can be expanded to study therapeutic efficacy of SEBPE against other health conditions.

## CRediT authorship contribution statement

**Faiqa Chaudhry:** Writing – review & editing, Writing – original draft, Visualization, Validation, Methodology, Formal analysis, Conceptualization. **Muhammad Laiq Ahmad:** Writing – review & editing, Writing – original draft, Visualization, Validation, Resources, Methodology, Investigation, Formal analysis. **Zafar Hayat:** Writing – original draft, Visualization, Validation, Software, Methodology, Formal analysis. **Muhammad Wasim Sajid:** Writing – review & editing, Visualization, Validation, Supervision, Software, Resources, Investigation, Formal analysis. **Muhammad Mustafa Qamar:** Writing – review & editing, Visualization, Software, Resources, Methodology, Formal analysis, Conceptualization. **Zunaira Basharat:** Writing – review & editing, Visualization, Validation, Resources, Methodology, Investigation, Formal analysis. **Muhammad Rizwan Tariq:** Writing – review & editing, Visualization, Validation, Software, Resources, Methodology, Investigation, Formal analysis, Data curation. **Amna Bibi:** Writing – review & editing, Software, Resources, Project administration, Methodology, Formal analysis, Data curation, Conceptualization. **Tawfiq Alsulami:** Writing – review & editing, Visualization, Validation, Supervision, Methodology, Investigation, Funding acquisition, Formal analysis, Data curation. **Suleiman A Athawab:** Data curation, Formal analysis, Investigation, Validation, Writing – review & editing. **Robert Mugabi:** Writing – review & editing, Validation, Software, Resources, Methodology, Formal analysis, Data curation. **Basim M. Alohali:** Conceptualization, Methodology, Software, Validation, Writing – review & editing. **Gulzar Ahmad Nayik:** Writing – review & editing, Visualization, Software, Resources, Methodology, Investigation, Formal analysis, Data curation.

## Declaration of competing interest

The authors declare that they have no known competing financial interests or personal relationships that could have appeared to influence the work reported in this paper.
